# Human resource management in the Georgian National Immunization Program: a baseline assessment

**DOI:** 10.1186/1478-4491-5-20

**Published:** 2007-07-31

**Authors:** Laura C Esmail, Jillian Clare Cohen-Kohler, Mamuka Djibuti

**Affiliations:** 1 Leslie Dan Faculty of Pharmacy, University of Toronto, Canada; 2 Curatio International Foundation, Tbilisi, Georgia

## Abstract

**Background:**

Georgia's health care system underwent dramatic reform after gaining independence in 1991. The decentralization of the health care system was one of the core elements of health care reform but reports suggest that human resource management issues were overlooked. The Georgian national immunization program was affected by these reforms and is not functioning at optimum levels. This paper describes the state of human resource management practices within the Georgian national immunization program in late 2004.

**Methods:**

Thirty districts were selected for the study. Within these districts, 392 providers and thirty immunization managers participated in the study. Survey questionnaires were administered through face-to-face interviews to immunization managers and a mail survey was administered to immunization providers. Qualitative data collection involved four focus groups. Analysis of variance (ANOVA) and Chi-square tests were used to test for differences between groups for continuous and categorical variables. Content analysis identified main themes within the focus groups.

**Results:**

Weak administrative links exist between the Centres of Public Health (CPH) and Primary Health Care (PHC) health facilities. There is a lack of clear management guidelines and only 49.6% of all health providers had written job descriptions. A common concern among all respondents was the extremely inadequate salary. Managers cited lack of authority and poor knowledge and skills in human resource management. Lack of resources and infrastructure were identified as major barriers to improving immunization.

**Conclusion:**

Our study found that the National Immunization Program in Georgia was characterized by weak organizational structure and processes and a lack of knowledge and skills in management and supervision, especially at peripheral levels. The development of the skills and processes of a well-managed workforce may help improve immunization rates, facilitate successful implementation of remaining health care reforms and is an overall, wise investment. However, reforms at strategic policy levels and across sectors will be necessary to address the systemic financial and health system constraints impeding the performance of the immunization program and the health care system as a whole.

## Background

Public health systems require effective human resource management for quality health system performance [1]. How well providers deliver services to patients depends on the processes that define, deploy and organize the workforce [2]. In any sector, the workforce must be motivated, well-staffed and appropriately skilled to do their job well [1]. This is particularly true for the health sector. Despite the importance of human resources to health care services, the health sector reform that took place in the 1990s failed to adequately address human resource issues [1]. Instead, reforms focused on areas such as cost-effectiveness, decentralization, privatization and reducing the role of government provision and financing of health care [3].

Decentralization is often a core component of health care reforms, however delegation of delivery of services may occur without delegation of adequate funding, institutional and administrative capacity, or the know-how to operate in and manage within the new health care structure [4]. In the context of rapid and dramatic reforms, a failure to address human resource management can easily jeopardize the success of any policy.

Georgia initiated efforts to implement health care reform in 1995. The reform's key components were fairly standard and included decentralization, privatization of health care services, the development of social insurance and contracting out for health care providers [5]. Reports suggest that the reforms were neither well-implemented nor comprehensive enough [6]. The decentralization of power to local municipalities was fragmented and the delegation of lines of responsibility was unclear [6]. Human resource management is one of the key barriers to successful health care reform in Georgia [5].

Reforms in the health care sector included efforts to improve the National Immunization Program. As we discuss, Georgia has scaled up its vaccination coverage since 1995, a critical component to achieving the Millennium Development Goal (MDG) of reducing child mortality by two thirds by the year 2015. More recent coverage rates in Georgia suggest improvements must still be made. Estimates in 2003 obtained from Georgia's new Immunization Management Information System (MIS) report coverage rates of 75% for DPT-3 and Polio-3, 48% for Hepatitis B-3 and 82% for Measles-1. Many variables can cause poor rates of immunization including inadequate financing, poor vaccine quality, poor vaccination practices, and weak health care systems [7] but one of the most common general barriers to improving immunization rates is human resources and management [8].

In our paper, we examine human resource management within the context of the National Immunization Programme in Georgia. Specifically, we explore the perceptions of managers and immunization providers in primary health care about existing management practices and processes. This research was carried out as part of a larger research project funded through Canada's International Development Research Centre (IDRC), which is examining the implementation and effectiveness of a model of supportive supervision in improving performance of the immunization program at the district level in Georgia. We hope our findings will contribute to an emerging literature in health system human resource management that is related to vaccine service delivery.

We organize our paper as follows. First, we introduce the immunization program in Georgia. Second, we describe our methodology. Third, we highlight the baseline results of our study, which focus on perceptions of management in the vaccine area. We conclude with a discussion of the findings, their generalizability and the limitations of our study.

### The Georgian National Immunization Programme

Preventative public health services are the responsibility of the Ministry of Health, Labour and Social Affairs (MoHLSA) [9]. The MoHLSA manages 12 regional Centres of Public Health (CPHs) across the country, which in turn oversee 54 smaller administrative CPHs. CPHs are responsible for implementing public health activities and the immunization program, collecting and analysing health statistics, and planning response measures and activities. In each district CPH, approximately one immunization manager is responsible for supervising the implementation of the immunization program, which includes vaccine procurement and distribution; maintenance of the cold chain; implementing the immunization management information system (MIS); and monitoring and supervision of primary health care providers for immunization-related issues. Primary health care workers provide immunization services at primary health care centres, which include large polyclinics and smaller ambulatory clinics. There is an average of 20–30 primary health care providers per district. Overall, there are approximately 100 immunization managers and 2500 primary health care providers involved in the implementation of the immunization program in Georgia.

Health care reforms of the 1990s failed, unfortunately, to improve the overall quality of the health care system and have even contributed to further health inequalities [9]. Some primary health care facilities are short of basic equipment and high utility expenses make it difficult for facilities to be maintained; municipal financing only covers current and not capital expenses [9,10]. Professional incomes have fallen dramatically since the reforms [10]. Physician incomes often are below official poverty levels; therefore many supplement their salaries by charging patients informally, a common practice in many transition countries. Rising out-of-pocket expenditure has limited the population's access to health care services, as many individuals avoid seeking health care until their condition is severe [11]. This also has the undesirable consequence of focusing the health system on curative rather than preventative health care services.

As we explain, sound human resource management practices are necessary for successful health care delivery in Georgia and are also vital to successful implementation of health care reforms [12]. Weak management is a common problem in many countries in Central and Eastern Europe and our study hopes to shed some light on management practices within the immunization program in Georgia in 2004 and areas for improvement.

### Research objective

The objective of our research is to examine the perceptions of primary health care workers concerning management processes and practices and organizational barriers within the immunization program in Georgia. This research is part of the baseline assessment of a broader study which assesses the impact of a supportive supervision intervention in improving human resource management practices and performance in the Georgian national immunization program at the district level in Georgia.

## Methods

### Research design

This study is the baseline assessment prior to intervention within a pre-post, quasi-experimental research design. We used a mixed methodology with focus groups and a quantitative survey. We defined human resource management broadly as "...the different functions involved in planning, managing and supporting the professional development of the health workforce within a health system..." [13]. We selected variables of interest guided by the study objectives and existing instruments, taking into account those which would be relevant to the Georgian context. These variables included work organization (which includes work environment, management and supervision processes and practices), roles and responsibilities (which includes job descriptions and understanding of roles and responsibilities), motivation and incentives. More details on the process of selection of these variables are described below under 'Data collection instruments'.

Prior to conducting the research, ethical approval was obtained from the Ethical Committee of the State Medical Academy, Tbilisi, Georgia and from the Ethics Review Office, University of Toronto. Informed consent was obtained from all participants before study implementation. We assumed that non-respondents of the baseline survey indicated a refusal to participate. No follow-up on reasons for refusal to participate was made.

### Sampling and sample sizes

For the intervention group, fifteen districts were randomly selected out of Georgia's 66 districts matched with another fifteen control districts which were selected by immunization performance indicators, geographical region and population density to the intervention districts. For the purposes of the analysis as presented in the manuscript, the two samples (i.e. intervention and control) were pooled. In all thirty districts, we selected one immunization manager from the local CPH (as proposed by the CPH) and randomly selected 20 health care providers working at immunization points at district polyclinics and village ambulatories (PHC facilities) who are directly responsible for rendering immunization services to the population. We used simple random sampling based upon a list of primary health care providers. Thus, the total proposed sample size was 600 primary care doctors/nurses and 30 immunization managers in the selected 30 districts. For the purpose of clarity, we refer to primary care doctors and nurses as 'immunization service providers' and CPH managers as 'immunization managers'.

### Data collection instruments

#### Surveys

We developed a survey after our literature review found no appropriate instruments for the study and its context. We adapted questions from the Management Sciences for Health's Human Resource Management Assessment Tool and other instruments used in health system assessments in Georgia [14,15]. First, we selected items that characterized aspects of human resource management, keeping the study objectives and the Georgian context in mind. Second, we held a discussion with a small group of immunization service providers and managers to obtain feedback on the survey and what topics might be more important considering the local context. We included topics only if consensus was reached. Then, the surveys were pre-tested among five immunization managers and five immunization service providers. Respondents were asked whether the questions were clear, relevant and whether they understood the context. Based upon their feedback, we revised the questionnaire for clarity. Through these processes, the investigators assessed the instruments' face and content validity. The general themes included in the survey were work organization, roles and responsibilities, supportive supervision, local governance and barriers to immunization. In this paper, we focus on work organization and roles and responsibilities.

#### Focus Groups

Focus groups were structured to fill in gaps and obtain in-depth information on baseline human resource management within the national immunization program. Four focus groups were conducted among immunization managers (CPH Directors and Managers) and immunization service providers (Health Facility Heads and Providers). We developed separate instruments for managers and providers to guide focus group discussions. We based the development of the focus group guides on the instruments mentioned above and the supportive supervision intervention. The guides were pilot tested and then revised based upon feedback from participants. We probed participants on the following topics: work organization, motivation and incentives, supportive supervision and performance of the immunization program. While we focus here on work organization, motivation and incentives, results address issues well beyond these themes.

### Data collection

#### Surveys

Survey questionnaires were administered to immunization managers and immunization service providers in the intervention and control districts between August and October 2004. The questionnaires were administered through face-to-face interviews to all thirty immunization managers. For the 600 providers, a mail survey was administered. Short questionnaires and informed consent forms were put in the envelope with post stamps and return address, which were distributed among selected participants. A five point Likert-scale was used to assess the degree of agreement with statements regarding human resource management. Confidentiality of all respondents was maintained through the replacement of personal identifiers with identification codes.

#### Focus groups

To ensure a range of opinions, researchers selected participants based upon their role in CPH management or PHC facility, size of district or facility and performance of district as informed by immunization indicators. In total, four focus groups were held with 8 immunization managers (4 CPH office directors, 5 CPH immunization managers) and 12 immunization service providers (5 health facility heads and 7 providers) in November 2004. Focus groups with managers ranged from 2 to 2.5 hours and from 1 to 1.5 hours with providers. Two people conducted each focus group: a moderator who led the discussion and a facilitator who handled logistics and took notes. The facilitator recorded the personal characteristics of the members making up the focus group and the time, duration, and location of the focus group. Discussions took place in a private setting, with minimal disruptions to allow people to feel they could voice their opinions freely. Focus groups were audio taped and detailed transcripts were prepared, stripped of identifiers and then coded. Notes and quotations were translated into English.

### Data analysis

#### Survey data

Descriptive statistics and between-groups comparison were done using SPSS software. The chi-square test was used to compare the categorical variables, and ANOVA to compare continuous variables. All indicators were measured and analysed at the individual level.

#### Focus groups

Preliminary codes were prepared prior to the focus groups, based upon the research topics. Upon transcription, two separate researchers reviewed the text and revised the codes. The transcripts were then coded and themes were deduced from the data.

## Results

Tables 1 and 2 present a basic description of the sample. The response rate among providers was 65% (intervention: 197 of 300; control: 195 of 300). Demographic and employment characteristics were similar among respondent and non-respondent providers. There were no refusals to participate in the study among immunization managers. Demographic characteristics are illustrated in Table 1. The majority of participants were female. No significant differences in mean age or mean years of professional experience among managers were found. Providers in the control districts were older and had more experience working in the current profession than those in the intervention district. Most managers had been trained as epidemiologists or health care managers (Table 2). Providers were mostly internists, paediatricians and family physicians. Providers were located in both urban (n = 236) and rural (n = 150) areas whereas all immunization managers (n = 30) were located in urban areas.

**Table 1:** Characteristics of Study Sample

**Immunization Managers**	**(N = 30)**
Proportion of females	80.0%
Mean age (SD)	42.8 (8.7)
Mean years in current profession (SD)	4.8 (2.3)
**Immunization Service Providers**	**(N = 392)**
Proportion of females	95.9%
Mean age (SD)	45.6 (9.4)
Mean years in current profession (SD)	19.8 (10.2)

**Table 2:** Educational Background of Participants

Training	Number
Immunization Managers	
**Health Care Manager**	**9**
**Epidemiologist**	**17**
**Parasitologist**	**1**
**General Practitioner**	**1**
**Pediatrician**	**2**
Immunization Service Providers	
**Internist**	**33**
**Pediatrician**	**152**
**Family Physician**	**34**
**Nurse**	**171**
**Gynecologist**	**1**
**Unknown**	**1**

Table 3 presents results of the descriptive analysis of survey responses provided by immunization managers. Responses suggest that managers find the work environment, its organization and management/seniority levels as adequate for their staff. However, when asked about specific barriers to the organization of work, they recognized the lack of management format and mandate, resource constraints, and financial and professional motivation as barriers. Managers did not seem to think that their own management capacity was an issue. We analysed responses for differences based upon geographic location, gender and age. Significantly more immunization managers in urban areas agreed that managers do not have the time to organize work well (mean = 3.20) compared with immunization managers in rural areas (mean = 1.96) (p = 0.001).

**Table 3:** Immunization Managers' Perception of Work Organization

**Overall organization of work (in CPH facility)**	Mean (95% CI)
1. I am satisfied with organization of work at my facility.	3.73 (3.46–4.01))
2. The overall work environment is very good at my facility.	3.33 (2.96–3.70)
3. My organization has sufficient authority to organize work so that subordinate staff is satisfied.	3.60 (3.25–3.95)

**Barriers to effective organization of work**	**Mean (95% CI)**

4. There are no barriers to organizing the work.	2.07 (1.97–2.16)
5. There is no clear format for managing/supervising health facilities and providers.	3.50 (3.19–3.81)
6. Health providers do not recognize the importance of better management and receiving supervision.	2.83 (2.44–3.23)
7. The supervision to health facilities/providers is not clearly mandated.	3.73 (3.41–4.06)
8. There is no penalty for managers if employees' performance is low.	4.37 (4.18–4.55)
9. Immunization managers do not have the time to organize work well	2.17 (1.87–2.46)
10. Immunization managers do not have the resources to organize work well.	4.10 (3.85–4.35)
11. Immunization managers do not have enough capacity to organize work well.	2.77 (2.48–3.06)
12. Immunization managers do not have the willingness to organize work well.	1.97 (1.81–2.12)
13. Immunization managers do not have the financial motivation to organize work well.	4.07 (3.81–4.32)
14. Immunization managers do not have the professional motivation to organize work well.	2.33 (2.05–2.62)

Note: (N = 30)

(5-point Likert Scale: 1 = strongly disagree, 5 = strongly agree)

Providers' responses illustrate a similar picture (Tables 4 and 5). Responses did not acknowledge organizational or management problems, however resource constraints were recognized. Table 5 shows that approximately half of all providers surveyed report having a written job description, while almost all respondents reported understanding their roles. Response rates varied, for individual questions, from 62% to 65%. There were no significant differences between respondents and non respondents in age, gender or duration of working in the current specialty. There were no significant differences found when comparing responses from urban and rural facilities.

**Table 4:** Immunization Service Providers' Perceptions of Work Organization

**Overall organization of work**	**Mean (95% CI)**	**N**
1. There is poor organization of work at my facility.	2.47 (2.37–2.57)	N = 385
2. There is lack of effective management and supervision from upper levels (both health facility and CPH).	2.55 (2.45–2.65)	N = 383
**Barriers to effective organization of work**		
3. Immunization managers do not have the resources to organize work well in the facility.	2.78 (2.68–2.88)	N = 380

Note: (5-point Likert Scale: 1 = strongly disagree, 5 = strongly agree)

**Table 5:** Number of Immunization Service Providers with job descriptions and understanding of job expectations

**Question (Y/N)**	**% Yes**
1. Do you have a written job description?	49.6 (n = 183, N = 369)
2. Do you know/understand what roles and tasks you must carry out in your job?	98.7 (n = 383, N = 388)

### Focus group discussion results

The main themes that emerged from the data addressed the organization of the immunization program, support and feedback, mechanisms for management and supervision, capacity and knowledge to manage and supervise, work motivation, and barriers relating to the health system and immunization. These themes are described in more detail below.

### Structural relationships and lines of responsibility

Immunization managers characterized the organization of the immunization program as extremely poor and chaotic. Respondents felt that there was a lack of clear delineation of organizational structure and lines of reporting. Managers cited weak administrative links between the CPH and health care facilities, making management of facilities and supervision of providers very difficult.

"Nobody knows who is responsible for human resource management in the health facilities. The doctor is appointed by the head of the policlinic, and the head of the policlinic is appointed by the Ministry of Property Management. We have minimal say in this process."

- Immunization Manager

Managers also viewed the reforms on health care facilities as confusing the lines of responsibility. Health facilities are now funded through different sources, including a federally-owned insurance scheme, but the CPH remains responsible for implementation of the immunization program.

"Doctors do not consider their managers as the CPH. Instead, they believe that the insurance company is responsible for everything because they cover all expenses."

- Immunization Manager

### Support and feedback from upper levels of management

Neither immunization providers nor managers were optimistic about the impact of the health care reform on their jobs. Providers stated that they do not receive enough support or feedback from their supervisors. Many providers expressed feelings of being left alone to solve complex problems such as issues related to poor working conditions, lack of equipment and lack of finances to repair infrastructure. They expressed a lack of support for issues relating to complex patient cases as well.

"We are self governors; we take care of our own. We are alone in doing repairs purchasing equipment...nobody helps us in persuading the parents or dealing with false contraindications."

- Rural Immunization Service Provider

Some CPH staff expressed similar views regarding upper levels of management. They viewed decentralization as being a key component of the problem.

"Management mechanisms should be strengthened at our level. At the district level, we always review the epidemiological situation including immunization coverage rates and always submit reports to the central level. However, feedback and response from the centre is very poor."

- Immunization Manager

### Lack of format for management and supervision

A common theme cited by immunization managers was a clear absence of guidelines or procedures describing management procedures. No mandates or regulations exist that delineate measures for human resource management or for supervision of health providers and health facilities. Providers do not have individual job descriptions and cited the lack of clear job expectations as a problem. They have monthly work plans that they review with the head of the health facility to discuss what has been accomplished. Providers have job contracts but they are vague and are not explicitly aware of their rights and responsibilities.

"Personnel knows by heart what their duties are and they follow their past experience and old traditions."

- Immunization Manager

Immunization managers described a disorganized human resource management system, characterized by a lack of procedures for monitoring, evaluation and performance incentives.

"There are some problems with monitoring the immunization program. The program has introduced some indicators, which should allow evaluation of providers' performance with implications on defining their salary, however currently nobody cares about these indicators. The insurance company created this indicator but did not explain how this indicator should work."

- Immunization Manager

In terms of incentives for improved performance, providers and supervisors reported few alternatives. Prior to reforms, penalties for poor performance were in place, however this is no longer the case. The only mechanism to discourage poor performance is a verbal or written warning. Some managers see the absence of penalties as negatively impacting providers' sense of responsibility and performance. Others claimed that no criterion exists for identifying good performance, despite the quantitative indicators mentioned above. Respondents were open to the potential of improved management and supervision on program performance.

### Human resource management capacity and authority

Providers (health facility heads) and immunization managers stated that no one has received any formal supervision or management training and respondents reported poor knowledge and skills in this area. Furthermore, respondents were not acquainted with the concept of supportive supervision.

"Lack of knowledge on how to manage or supervise could be one of the reasons for insufficient management and supervision, because training on these issues was not provided to the CPH staff."

- Immunization Managers

When asked about potential barriers to organizing work well, respondents did not see time as a barrier, but concerns were raised about adequate human resources and financial resources to cover increased supervisory tasks and visits that would accompany the implementation of supportive supervision. Notably, immunization managers viewed management problems as related to a lack of authority on their part rather than inadequate management knowledge and skills. Managers blame decentralization for this problem. Previously, they had more control over tasks such as creating job vacancies, and hiring or dismissing employees and could impose penalties in cases of poor performance. Now, they are restricted in their ability to improve the working conditions, hire employees and penalize providers.

### Job incentives and motivation

A major concern raised by all respondents was low salary levels. Immunization managers and providers emphasized their salaries were incommensurate with the scope of work they were required to do. Also, managers identified low provider motivation as affecting quality health service delivery.

"I know, in case of the improvement of the quality of my work and receiving an excellent evaluation, it will not be reflected in the financial incentives."

- Immunization Provider

When specifically asked, providers and managers cited non-financial sources of motivation as well. They cited factors such as an increased sense of responsibility, the opportunity for professional improvement, seeing positive results and getting feedback and attention from senior management. However, these alternative sources of motivation did not seem to outweigh the importance of having an adequate salary.

### General health system and immunization-specific issues

While the focus group topics centred on management procedures and practices, respondents emphasized other barriers to the performance of the immunization program. The most common reasons cited across all focus groups were negative media coverage about the potential adverse effects of vaccination, a low awareness in the population about the benefits of vaccination, and neurologists advising their patients against vaccination. In addition, immunization managers cited problems of inadequate knowledge among providers with respect to vaccine prescribing. Respondents emphasized increased financial resources as key to improving immunization program performance by helping to address some of these deficiencies.

The lack of financial resources results in problems ranging from low salaries to infrastructure and equipment in disrepair. For example, one immunization provider reported that she occasionally purchased pharmaceuticals for her patients from her own salary. Other issues include unreliable electricity and lack of heating in some villages. Some facilities lack refrigeration devices.

"There are villages with electricity for only 3–4 hours a day. Some clinics do not have fridges for vaccines."

- Immunization Manager

These unreliable conditions cause reluctance by some physicians to administer or prescribe vaccines. Financial problems limit managers' ability to visit and communicate with remote areas and again, anecdotal reports suggest some providers may pay out of pocket for taxi fares required to obtain vaccines from the CPH.

## Discussion

The findings of this study are based on the human resource management structure and practices within the Georgian National Immunization Program in late 2004. While our study does not draw a direct link between the poor performance of the immunization program to weak human resource management, it is clear that improvements in this area are needed and subsequently, improvements may very well result in a positive effect on performance.

Our results identify many areas for improvement, starting with the organization of work. The weak structural relationships and unclear lines of responsibility found in this study support the findings of Hotchiss et al. who found similar issues in the Imereti region of Georgia [15]. Decentralization often results in confused lines of reporting and this can adversely affect accountability and staff motivation [16]. The scenario, where human resource management is not effectively integrated as part of the reforms, is widespread [1,2,4] and similar to that experienced in countries in the CEE/NIS [10]. Ideally, appropriate consideration of human resource management should occur during, or immediately after, the decentralization process [17]. Implementing these HRM reforms after the fact is necessary but will be more difficult, especially if the Ministry of Health no longer has the authority or capacity to implement the necessary changes [4].

To facilitate the organization of work, CPH managers must have sufficient authority to manage their workforce and take the requisite steps to ensure health targets are met [5]. Decentralization often results in increased responsibilities for health care delivery but fails to delegate the necessary autonomy to determine health care budgets or hire and fire staff. The delegation of even minimal control over resource allocation and staffing decisions can result in positive improvements since managers can facilitate some improvements quickly without having to continually access upper levels of management [16]. Managers linked their lack of authority to their incapacity to penalize poor provider performance.

Planning and human resource management skills generally do not exist at local, peripheral levels in developing countries [18]. This is likely the case across much of the CEE/NIS region, given the pre-reform system, which was a highly centralized system with little responsibility at local levels [10]. Training towards these new skills requires capacity and resources [17], which is often lacking and was the situation during implementation in much of the CEE/NIS [10]. Processes for HR management such as setting salaries, recruitment, performance assessment and staff discipline must be defined clearly and explicitly, in conjunction with a system to train staff in the use of these processes [17].

With regard to the providers' work environment, our results show that providers do not feel adequately supported in their work. The nature of supervision that they receive is important; punitive supervision or supervision that seems to mimic "sterile administrative procedures" can sometimes have negative effects on provider motivation and performance [16]. Supervision becomes that much more important in decentralized systems, where new skills and competencies are needed and clear and open lines of communication are critical to ensure a coordinated and efficiently functioning health care system [16]. CPH staff members' lack of knowledge and skills in supportive supervision suggest that there is room for improvement in this area and that this might have a positive impact on provider motivation.

In the context of health system infrastructure, an adequate work environment is key to effective delivery of health care services and can actually improve worker motivation [19]. Poor infrastructure, lack of supplies, intermittent electricity and heating and interruption of the cold chain are all factors that can impede an effective immunization program and worker motivation. Improved human resource management may open the lines of communication and facilitate raising these concerns at the appropriate authority level. The Government of Georgia is presently implementing a health care reform initiative, with a focus on improving infrastructure, provision of equipment and training family doctors and family practice managers. Hopefully, these efforts will ameliorate health system issues and facilitate more significant improvements in immunization rates. Underlying these system-wide issues is the problem of inadequate financing. Municipalities have inadequate budgets and cannot cover capital expenses. The delegation of authority for revenue collection to the municipalities is slow and they still heavily rely on transfer payments from central government, which is also sluggish in its approach [5].

Our study illustrated the lack of clarity that managers and providers have with respect to their roles and responsibilities. Immunization managers emphasized a lack of clear guidelines about how to perform their jobs well and only half of providers reported having written job descriptions. Again, these aspects are often overlooked in the process of decentralized reform. The delegation of human resource management must accompany revision of organizational structures, reporting relationships, and job descriptions [17].

The study cites many factors that could contribute to low provider motivation not the least of which is low salary, a widespread problem in Georgia. Martinez and Collins report that competitive salaries and the "means to do work" are essential pre-requisites to improving staff performance and that evidence suggests that interventions without these components in place are ineffective [20]. The severe context of unemployment in Georgia may complicate these findings since health care workers may be afraid of losing their jobs. However, anecdotal reports suggest that providers in Georgia attempt to find alternative jobs, either in the private sector, or other employment opportunities, which is commonly reported elsewhere [21]. Providing a sufficient salary will improve worker motivation; innovative ways to increase salaries of health workers in resource-constrained settings should be considered, one of which includes government prioritization of certain key sectors for wage increases [16].

Underpayment can contribute to poor staff motivation but a poor working environment and minimal opportunities for advancement or learning can exacerbate the problem [20]. Dieleman's study in Vietnam showed that appreciation by managers, colleagues and the community were encouraging factors [19]. In the context of Georgia, Bennett and Gzirishvili consistently found hospital workers emphasizing the "importance of social relationships between workers" [6]. It is plausible that these social relationships would gain importance in the context of the socioeconomic transition currently present in Georgia, however they are unlikely to be enough to compensate for an adequate salary.

Results should be considered in the context of the study's limitations. First, the study did not follow a pre-existing conceptual framework, which may limit the comparison of results to other research. However, it is hoped that study will provide a baseline picture of deficiencies within human resource management in Georgia, and identify areas for future research. Second, evidence on the validity and reliability of the Likert-scale surveys is limited but the consistency of focus group results with survey responses provides additional evidence supporting the validity of the Likert-scale surveys used. Third, reporting bias may have confounded some of the participants' responses, especially during focus groups where perceptions were shared in the presence of other participants. Still, other studies and reports cite similar issues raised here [5,6,12], suggesting that the results are externally valid. For example, in Hotchiss' evaluation of an intervention to improve disease-surveillance and response activities, they found that many health system barriers limited the intervention's effectiveness and noted 'weak accountability relationships' and unclear roles and responsibility across levels of the health care system [15]. Also, Afford's review of the challenges facing health workers in Central and Eastern Europe and the newly independent states describes the impact of reforms in reducing the state's role, disrupting previous structures for managing performance, staff and delegating authority to unprepared peripheral levels [10]. The implications of our findings suggest that interventions are needed at policy and strategic levels to address organizational issues as well as training programs at the local levels to enhance human resource management capacity. Issues relating to financial constraints, infrastructure and poor working environment must be addressed to facilitate gains made by organizational and managerial improvements and will require a multi-sectoral approach.

## Conclusion

The results of this study suggest that in 2004, the National Immunization Program in Georgia was characterized by poor work organization, a variable work environment, and weak management structures and practices, especially at peripheral levels. The development of the structures, processes and skills of a well-managed workforce may help improve immunization rates, facilitate successful implementation of remaining health care reforms and is an overall, good investment. However, reforms at strategic policy levels and across sectors will be necessary to address the systemic financial and health system constraints impeding the performance of the immunization program and the health care system as a whole.

## Competing interests

The author(s) declare that they have no competing interests.

## Authors' contributions

All authors contributed to the conception, design, and interpretation of the study. LE conceived, drafted and finalized the manuscript. JCK contributed to the conception of the manuscript and drafting and finalization of the manuscript. MD contributed to the implementation of study in Georgia and comments on the manuscript.
